# Neighbour sensing through rhizodeposits in sorghum affects plant physiology and productivity

**DOI:** 10.1093/aobpla/plaf065

**Published:** 2025-11-13

**Authors:** Shiran Ben-Zeev, Amanda Penn, Erica H Lawrence-Paul, Desa Rae Abrams, Rotem Ben-Zeev, Carolyn Lowry, Jesse R Lasky

**Affiliations:** Department of Biology, Müller Laboratory, Curtin Road University Park, PA 16802-5301, United States; Department of Biology, Müller Laboratory, Curtin Road University Park, PA 16802-5301, United States; Department of Biology, Müller Laboratory, Curtin Road University Park, PA 16802-5301, United States; Department of Biology, Müller Laboratory, Curtin Road University Park, PA 16802-5301, United States; Department of Biology, Müller Laboratory, Curtin Road University Park, PA 16802-5301, United States; Department of Plant Science, Tyson Building University Park, PA 16802, United States; Department of Biology, Müller Laboratory, Curtin Road University Park, PA 16802-5301, United States; Plants, Ecosystems & Climate

**Keywords:** leachate, plant–plant interaction, rhizodeposits, sorghum

## Abstract

Plant–plant interactions play a crucial role in shaping the growth environment for crops, impacting their productivity and resilience to stress. Interactions between plants have been incorporated into breeding programmes by selecting new target traits that will advance plants’ abilities to produce in high densities. The study of plant–plant interactions belowground promises new pathways and traits for crop improvement. This study focuses on the developmental and physiological responses of sorghum (*Sorghum bicolor* L.) genotypes to neighbouring sorghum plants. In this study, we used two growing methods: (i) a focal plant surrounded by neighbouring plants in the same pot but without shading, and (ii) a focal plant grown either alone or surrounded by neighbours, irrigated with nutrient solution that was passed through pots (leachates) with or without plants. Our results show that the presence of neighbours in the same pot led to reduced dry weight, plant height, and leaf area of the focal plant. In addition, the presence of neighbours reduced stomatal conductance and photosystem II quantum yield. While the response direction was similar across tested genotypes, the magnitude varied. The results were repeated when neighbouring plants were not grown in the same pot, but a nutrient solution was passed through the root systems of other plants into a separate pot containing another plant. Furthermore, we saw a reduction in assimilation rate and stomatal conductance when plants were exposed to either the physical presence of neighbours or leachate. We did not find differences in root architecture in either treatment. These results show that plants change their growth in response to neighbours and that the signal is carried through the liquid phase of the soil. Our findings provide insights into sorghum plants’ responses to belowground signalling from neighbouring plants and lay the foundation for future studies enabling increased crop performance under high-density planting conditions.

## Teaser

Plants “talk” underground. In sorghum, we show that chemical signals released by roots (rhizodeposits) from neighboring plants reduce growth and photosynthesis, even when light and nutrients are not limiting. Using two greenhouse experiments that separated physical neighbors from their leachates, we found consistent decreases in biomass, leaf area, stomatal conductance, and carbon assimilation. Leachates alone reproduced most effects, indicating a liquid-phase signal that conveys the presence of neighbors. These results reveal a below-ground communication channel that can shape how crops perform at high planting densities. Recognizing and ultimately breeding for genotypes that are less sensitive to these signals could improve stand productivity and resilience under intensive agriculture.

## Introduction

The interactions between a plant and its neighbours define much of the immediate environment in which it grows. This environment can be beneficial or disadvantageous, depending on the identity of the surrounding plants, whether plant interactions result in competition or facilitation, and the plant’s ability to tolerate competition (Farrior 2014). Global climate change and the need to produce more food from less land require increased farming intensity. One approach to improving productivity has been developing crops that yield more at the stand level when grown in high densities rather than focusing on individual plant productivity. Since the 1960s, ideotypes—putatively ideal combinations of crop phenotypes—have been developed to reduce competition and enable more plants to grow per unit area (Donald 1968). Plants compete for sunlight aboveground by increasing leaf area and stem height. Thus, many traits have been targeted in breeding programmes, such as a more vertical leaf angle to avoid mutual shading and smaller tassels in maize (Sandhu and Dhillon 2021). Understanding the developmental and physiological response to neighbours and its underlying genetic basis could enable further breeding for higher performance under intense competition for future farming.

Competition between plants, by definition, leads to reductions in fitness (Casper and Jackson 1997, Aschehoug et al. 2016). Competition may become more severe when resources are limited in supply and availability (Callaway and Walker 1997). Plant–plant competition can decrease stand-level productivity when individual plants inhibit the growth of neighbouring plants through increased uptake of nutrients or water or through biochemicals affecting the germination, growth, or development of neighbouring plants (Putnam 1985, Bogatek et al. 2006). An opposite effect is facilitation among neighbours, e.g. by recruiting beneficial microbes, changing the pH, or changing soil structure in a way that benefits all plants in a stand (Farrior 2014, Henriksson et al. 2021). A recent study (Lorts and Lasky 2020) of competition without resource limitation demonstrated that *Arabidopsis* plants responded to neighbouring grasses via changes in aboveground (flowering time and rosette and silique number) and belowground (root diameter) traits, producing shorter inflorescences and reducing root diameter. The same study also showed that this response changes under abiotic stress, such as drought. These findings suggest that plants alter their development in response to neighbour presence even without resource limitation, raising a question of whether such responses are triggered by biochemical cues or signalling molecules. This idea is explored in our study.

While resource competition has been the primary focus of ideotype development, belowground signalling among neighbouring plants may also influence productivity (Bhowmik and Doll 1984), but is relatively less studied. Plants often respond to the presence of neighbours by altering their growth, and these responses can arise through distinct mechanisms. For example, shade avoidance is a well-known light-mediated response in which plants grow taller and reallocate biomass to shoots in response to low red-to-far-red light ratios (Earley et al. 1966, Page et al. 2011). In young maize plants, shading cues led to a lower root-to-shoot ratio and increased leaf area (Page et al. 2011). Plants may also respond to belowground resource competition by increasing their root system size or altering root placement to soil depths, or adjusting their root distribution to the proximity of a competitor’s root systems (Lepik et al. 2021).

Beyond responses to physical or competition cues, plants can also perceive neighbours through biochemical signalling. Several studies have reported changes in root gene expression, metabolites, and proteins in the presence of neighbours, suggesting plant–plant interaction responses on a molecular level (Schenk 2006, Afifi and Swanton 2011, Khashi et al. 2019). For example, Schmid et al. (2013) presented results from *Arabidopsis* plants, which responded to the presence of a neighbour plant in the same pot by up- or down-regulating genes involved in responding to parasitic fungi, salt, heat, hypoxia, and phosphorus deficiency. The same study also reported a reduction in shoot biomass and changes to root morphology in *Arabidopsis* plants interacting with the roots of *Hieracium pilosella.*

Responses to neighbours start at the seed stage (Hilhorst and Karssen 2000), leading to growth changes in seedlings, suggesting that root recognition precedes the aboveground encounters (Afifi and Swanton 2011) and sensing of neighbours (Schmid et al. 2013, Bilas et al. 2021). The presence of roots belonging to a different species drives changes in root distribution to different depths, similar to the changes caused by nutrient and water availability and placement (Callaway and Walker 1997, Du et al. 2021). In the rhizosphere, sensing of roots occurs via signals such as nutrient deficiency, water scarcity, and, importantly, chemical sensing of small recognition molecules and specific proteins secreted in root exudates (Badri et al. 2012). Lectins (e.g. jacalin), peroxidases, and jasmonic acid have been suggested as molecules mediating root responses to neighbours (including weed species in fields) (Bilas et al. 2021). Another important set of molecules affecting neighbouring plants’ growth regulation are strigolactones (SLs) (Proust et al. 2011, Al-Babili and Bouwmeester 2015). SLs are a class of plant hormones that have been shown to mediate interspecies plant–plant communication (Wheeldon et al. 2022) and parasite–host recognition (Babiker and Hamdoun 1983, Bellis et al. 2020, Mwangangi et al. 2023) and are involved in plant–plant interaction and development in the presence of neighbours (Bilas et al. 2021). The effect of root–root communication on flowering times has been reported in *Brassica napa* (L.), such that day-length sensing could be mimicked when exudates from plants sensing a long day are used to irrigate plants grown under short days, leading to significantly earlier bolting and flowering (Falik et al. 2014).

Sorghum (*Sorghum bicolor* L. (Moench)) is a C4 cereal grown in rain-fed and irrigated environments on high-yielding and marginal soils from Africa and the Middle East to India, Australia, and the USA. It is the fifth most economically important crop in the world (FAOSTAT, https://www.fao.org/faostat/en/#data), extensively grown in various sectors such as biofuel production, forage cultivation, and grain production. Domesticated in Africa (De Wet and Harlan 1971), where it is still grown on vast areas of land, sorghum is more resilient to environmental stress than many other crops, making it an important model species for studying the unique strategies for dealing with the drought stress of C4 grasses (Pardo and VanBuren 2021). One study on sowing densities in sorghum (essentially testing plant–plant interactions) found that sorghum harvest indices decreased when water was limited and that this reduction was uniform across sowing densities (Berenguer and Faci 2001). High sowing densities have also been shown to result in stand-level yield reduction across three drought treatments (Jahanzad et al. 2013), representing a possible negative effect caused by neighbours. Two other studies reported a lack of response or increased stand-level productivity under higher densities (Gerik and Neely 1987, Raphaël et al. 2024), indicating a variability in the response to density. Sorghum is known to have allelopathic traits, and growing sorghum has been suggested as a way to reduce weed populations and their biomass (Cheema and Khaliq 2000, Raza et al. 2021). A well-known allelopathic compound of sorghum is sorgoleone (Einhellig and Souza 1992), which has been shown to reduce photosynthesis and water uptake in different species (Einhellig et al. 1993, Hejl and Koster 2004) at concentrations of 50–100 µM. However, it is unclear if sorgoleone is exudated more under drought stress. Some evidence also exists for sorghum root exudates having autotoxic potential. However, those studies used very high concentrations of sorgoleone (10%–30% of the solution—mixed with distilled water), and root exudates were extracted in methanol, dried, and resuspended (Tibugari et al. 2020).

The overall goal of our study was to evaluate whether the effects of neighbours on sorghum plants’ growth and productivity can occur via belowground cues under conditions where resource competition is minimized. Specifically, we tested three hypotheses:

- The presence of neighbouring plants’ roots will lead to changes in root and shoot growth, plant physiology, and reduced productivity, even in the absence of resource limitations. However, the effects of neighbouring plants will be larger under water-limited conditions.
- Sorghum plants will grow taller in response to the presence of neighbours, which would promote light capture, even without sensing shading.
- Biochemical compounds present in leachates can trigger physiological and developmental responses to neighbours, thereby mimicking the negative effects of physical competition.

Our goal in these experiments was to observe the growth, development, root architecture, and physiology of sorghum plants in systems where the focal plants were allowed to interact with their neighbours through signalling in the rhizosphere while preventing aboveground shading (by forcing neighbour plants away from the tested focal plant). Further, we sought to isolate the effects of rhizodeposits, including root exudates and other biochemical compounds released into the soil by the plant (Duchene et al. 2017). To achieve this, we studied plants without physical neighbours in the same pot. We accomplished this by transferring leachates from source pots with ‘neighbour’ plants to pots of focal plants with and without the physical presence of neighbours (Supplementary Figure S1).

## Materials and methods

### Experiment 1: drought and physical interaction (EXP 1)

#### Plant material

Ten sorghum genotypes obtained from USDA GRIN were selected to represent a wide range of variation. They included improved genotypes important for US breeding: RTx430 and BTx623 (PI655996 and PI564163, respectively), well-studied lines: Shanqui Red (PI656025) and SRN-39 (PI 656027), and landraces from India, Sudan, Nigeria, and South Africa (Supplementary Table S1). These lines are referred to hereafter as focal plants for which data were collected. In addition, one common sorghum genotype (BTx3440), received from William L. Rooney at Texas A&M, was used as the ‘neighbour’ line across all experiments to avoid variation in signals and growth habits. The competitor line was selected for its high performance under drought conditions and seed availability.

#### Experimental design and growing conditions

Our goal in this experiment was to grow sorghum plants of a focal genotype with neighbouring plants in the same pot, allowing them to interact belowground but preventing mutual shading and competition for light. In addition, we tested the effect of drought on competition by including well-watered and water-limited treatments.

The experiment included four treatments in a full factorial randomized block design, including four blocks with one repetition of each genotype-by-treatment included in each randomized block. The treatments were well-watered without competition (NC_WW), well-watered with competition (C_WW), water limited without competition (NC_WL), and water limited with competition (C_WL).

The competition treatments were created by surrounding a focal genotype with 5 neighbour plants (equivalent to 16 plants per square meter plant density or ∼125 000 plants/ha), with the recommended range for forage sorghum sowing in the USA being 125 000–200 000 plants/ha in well-watered environments (https://extension.psu.edu/forage-sorghum-planting) (Supplementary Figure S1). To avoid the effects of shading by the neighbouring plants and focus our experiments on belowground interactions, the shoots of neighbouring plants were trained to grow away from the focal plant by placing a paperboard bowl with a hole cut in its middle point so that the focal plant grows through the bowl and competitor plants are forced to grow sideways. At a later stage of growing (∼25 days after sowing—DAS), neighbour plants were restrained toward the outside of the pot using a string to prevent them from shading the focal plant.

Seeds were sown in vermiculite in bottom-watered pots. Five days after sowing, seedlings were transplanted into 5.7 l pots (Supplementary Figure S1, left) filled with 80% potting mix (Pro-mix BX, PA, USA) and 20% Turface^©^ (Turface Athletics, IL, USA). To avoid any nutrient limitation, the soil in each pot was mixed with 14 g of Osmocote^©^ flower and vegetable 14-14-14 slow-release fertilizer (ICL, Israel), a high fertilizer rate selected to lead to high nutrient availability for the entire growth period. The dry weight was measured for each pot, and the volume required for saturation was tested and found to be 89% of the dry weight. At the start of the experiment, pots in the well-watered treatment were watered with the volume required for saturation. For the drought treatment, 75% of the saturation volume of water was added to allow seedlings to establish without saturating the soil. Pots were weighed every 3–4 days.

Drought treatment pots were maintained at a weight (soil + water) of 25%–35% of the weight required at saturation—once they had dried sufficiently (17 days after planting), and well-watered pots were maintained at 55%–70%. The water deficit was validated using three soil volumetric moisture probes for each treatment (ZL6 Basic, Meter Environment, WA, USA), confirming the weight measurements.

Plants were grown in a greenhouse with day/night temperatures set to 28/18°C, with added light (3000 lumens—38.92 µmol/s quantum photon flux) supplied by high pressure sodium lights to produce a 14-h day length for 35 DAS in each cycle. The first cycle started on 27 December 2022, and the last cycle was harvested on 23 May 2023.

#### Phenotype measurements

For the focal plant in each pot, height was recorded as the distance from the soil to the base of the top fully expanded leaf. Height was measured every 3–4 days to calculate the height growth rate. Stomatal conductance and chlorophyll fluorescence were measured using a Li-Cor 600 fluorometer porometer (Li-Cor, Lincoln, NE, USA). Measurements were taken between 11 a.m. and 12 p.m. under ambient conditions where the average light level was 567 µmol m^−2^ s^−1^, average RH was 21%, and average leaf temperature was 25.5°C. Stability for gsw (stomatal conductance to water vapour) was set to 0.005 mol m^−2^ s^−1^ for a period of 2 s and *F* to 5 s^−1^ for 2 s. At harvest, the height was recorded, and the leaf area was measured. For EXP 1, the leaf area was determined using a Li-Cor 2000 Plant Canopy Analyzer (Li-Cor, Lincoln, NE, USA). Shoot dry weight was measured using a bench-top scale after drying the plants in an oven at 60°C for 3 days.

### Experiment 2: leachate transfer (EXP 2)

#### Plant material

Two of the genotypes used in EXP 1 were selected based on their wide use in the literature and to represent modern elite lines—RTx430 and BTx623 (PI655996 and PI564163, respectively), along with the recurring neighbouring genotype, BTx3440.

#### Experimental design and growing conditions

Following the results from the physical competition experiment, we designed a second experiment to separate neighbouring plants’ physical and biochemical presence on focal plants. This was achieved by transferring the leachate, likely including rhizodeposits, from a source pot of neighbours to a target pot with a focal plant. The system included two sets of pots: source pots positioned above drain holes (funnels) on 30 cm high raised benches and target pots positioned next to source pots on the growth table below (Supplementary Figure S1). This experiment consisted of four treatments with one tested focal plant from genotype—RTx430 or BTx623L: Control—one focal plant grown as single plants in pots with the irrigation solution transferred through the media without plants in it. Competition—a focal plant grown in the same pot with three plants of the BTx3440 genotype (similar to EXP 1). Leachate—leachates from source pots with three BTx3440 plants flowing into funnels and through a PVC tube to the target pots. Competition + leachate—plants were grown with three competitor plants and irrigated by leachate. Plants in all pots received light from multiple directions, limiting the effects of shading. While some shading may have accrued based on the design of the experiment, all target pots were exposed to similar (low) levels of shading from plants of source pots from their own row or adjacent ones. The experiment was designed in three randomized blocks with two replicates of each genotype-by-treatment combination for the leachate treatment. To allow easy root cleaning for visualization, the growth media was 60% sand (pool filter sand, Quikrete, Atlanta, GA, USA), 20% Turface^©^ (Turface Athletics, IL, USA), and 20% clay (Kaolin calcinated clay, CB minerals LLC, Mamaroneck, NY, USA). Pots were determined to require ∼150 ml of water to be saturated. Thus, source pots were irrigated with 300 ml nutrient solution (Miracle-gro Aero Garden, 4-3-6 (NPK) + Mg and Ca, Liquid Plant Food—Miracle-Gro, Marysville, OH, at 2 ml/4 l), every 2 days to avoid water and nutrient stress. Resulting in an addition of 1.7, 1.3, and 2.6 ppm of N, P, and K, respectively, daily—which was expected to supply the necessary amounts for an entire season given in 1 month (Pennsylvania State University, Agricultural Extension Service). Water amounts passing through the source pots were measured weekly to validate that there were no differences in the amount of water applied to experimental pots between control and leachate treatments, and the percentage of water passing was consistently 65%–75% of the applied water. Target pots were only irrigated through source pots. Plants were harvested at 37 DAS.

#### Phenotype measurements

For the focal plant in each pot, the same phenotypes as EXP 1 were collected. In addition, detailed gas exchange measurements were collected on newly fully expanded leaves on three plants of each genotype-by-treatment combination using a LI-6800 Portable Photosynthesis System (Li-Cor, Lincoln, NE, USA). Measurements were taken at a PAR (photosynthetically active radiation) of 1200 µmol m^−2^ s^−1^, CO_2_ 420 µmol mol^−1^, flow 600 µmol s^−1^, Tair 25°C, and RH 50%. Measurements were taken when stability was observed for A and gsw after leaves were in the chamber for a minimum of 2 min, and stability measures for delta CO_2_ (slope limit 0.5) and δH_2_O (slope limit 0.1) were also met for a period of 20 s. A saturating light level of 1200 was determined using light response curves, and all reported values are averages for measurements taken every 10 s over a total of 2 min. Data were collected between 9 a.m. and 3 p.m., and the sampling order was randomized. These measurements included the carbon assimilation rate, stomatal conductance, and transpiration rate for three plants for every treatment by genotype combination, as well as instantaneous water-use efficiency calculated by dividing the assimilation rate by the transpiration rate (Farquhar and Richards 1984). At harvest (37 DAS), plants were removed intact from their containers, and roots were washed to remove soil. The roots of plants of the leachate transfer experiment (5 reps) were placed into sheet protectors (transparent bags) and scanned at 600 dpi using an Epson 600V scanner (Seiko Epson Corp., Nagano, Japan). Images were analysed using RhizoVision Explorer v2.0.3 for total root area, median root diameter, and number of root tips (Seethepalli and York 2020) using algorithms described by Seethepalli et al. (2021).

#### Statistical analysis

Linear mixed-effect models were used to estimate the effects of competition and drought. For EXP 1, the model included genotype, competition, and drought as fixed effects, along with their interaction, with block as a random effect. For EXP 2, the model included treatment and genotype as fixed effects along with their interaction, with block as a random effect, except for Licor 6800 measurements, which did not include the block effect since it used representative plants. The denominator degrees of freedom were estimated using the Satterthwaite approximations. We calculated the least square (LS) mean values using a model that included genotype, drought, and competition, as well as their interaction, as fixed effects and the experimental blocks as a random effect. Preliminary analyses, including Tukey Honestly Significant Difference (HSD) and LS means in JMP^®^ (SAS Institute, Cary, NC, USA, 1989–2024), were used to explore treatment effects and guide model selection before formal analysis in R. We compared the different models used for LS means in R (R Core Team 2025) using the lmertest package (Kuznetsova et al. 2015). We then used the ggplot2 package (Wickham et al. 2016) for plotting, and significance was calculated with the ggsignif package (Ahlmann-Eltze and Patil 2021). Correlations were calculated using the Corrplot package in R (Wei et al. 2017). The lsmeans package (Lenth and Lenth 2018) was used to calculate the cumulative fixed effects values for reaction norms analysis.

## Results

### Experiment 1

#### Neighbouring plants reduce performance equivalent to a water-limited treatment

**Figure 1.**
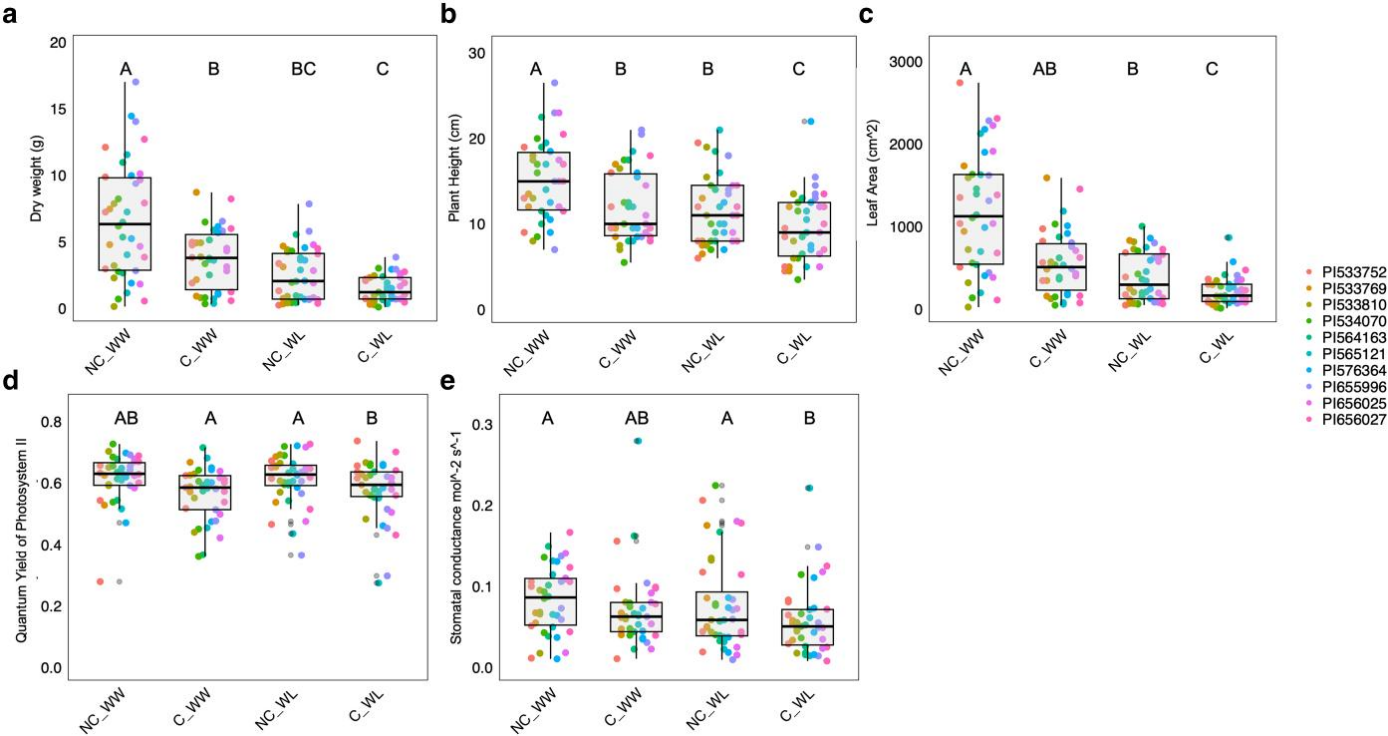
a—Shoot dry weight (g), b—plant height (cm), c—leaf area, d—quantum yield of photosystem 2, and e—ambient stomatal conductance, all measured 34 DAS. Values presented are all four reps of ten genotypes under water-limited (WL) and well-watered (WW) conditions, with competitor plants (Competition) and without (No Competition). Connecting letters represent significance in Tukey HSD tests between treatments (*P* values lower than .05).

Dry weight values were significantly reduced under competition in well-watered conditions (C_WW) (see Supplementary Table S2 for *F* ratios and in-depth details) compared to the no competition-well-watered treatment (NC_WW), despite the ample nutrient fertilization provided in the competition treatment pots. Dry weight was reduced in both water-limited (WL) treatments compared to WW treatments, with no significant difference between the WL competition (C) and NC (Fig. 1a; Supplementary Table S2). For dry weight, these values were 73%, 59%, and 43% lower than the control in the competition water limited (C_WL), no competition water limited (NC_WL), and C_WW treatments, respectively. Plant height at the end of the growth period (34 DAS) was similarly reduced in response to competition and water limitation, with a 20% reduction in C_WW and a 25% reduction in NC_WL treatments compared to the control. A further significant reduction was observed under the combined competition and drought—C_WL (40%) treatment (Fig. 1b; Supplementary Table S2).

Leaf area was significantly reduced in all treatments compared to the NC_WW control, with the NC_WL treatment being significantly lower than the control (65% reduction), and the C_WL treatment being significantly lower than both WW (82% reduction) and NC_WL. The C_WW treatment leaf area (55% reduction) was not significantly lower than NC_WW (Fig. 1c; Supplementary Table S2).

Similar to dry weight, competition led to a significant decrease in the quantum yield of photosystem II (PhiPSII) (Fig. 1d; Supplementary Table S2) under the WL treatment (representing an LS means difference of 8%) but not under the WW treatment. Stomatal conductance was lower under the competition treatment in both water treatments. A 25% reduction was found for the C_WL, respectively, compared to the control (*P* = .01, Fig. 1e; Supplementary Table S2).

#### Variation in competition response among genotypes and between water treatments

**Figure 2.**
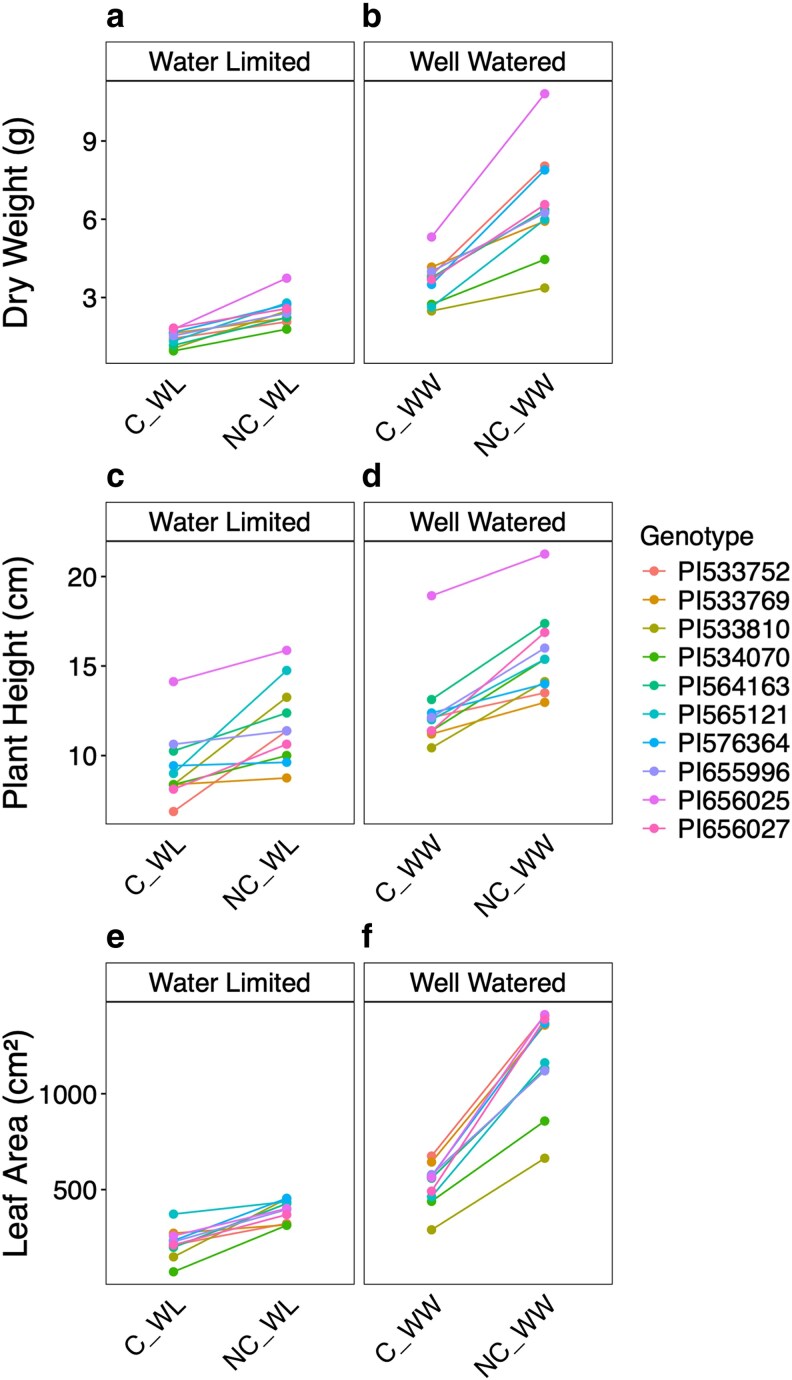
Reaction norms of the ten tested genotypes for dry weight (a, b), height (c, d), and leaf area (e, f) between the competition (c) and no competition (NC) treatment within the water-limited (WL, left), well-watered (WW, right) treatments. Values presented for each line are the LS means obtained from a model, which included the lines (PI numbers for each line are presented in Supplementary Table S1), competition, experiment, water treatment factors, and competition by genotype interaction.

We included genotypes from different regions and farming systems to test genotypic differences. However, the genotype effect was only significant for plant height, and none of the genotype-by-treatment interactions were significant (including for plant height), indicating similar responses to neighbours. This overall trend was generally uniform in direction (no significant genotype × competition or genotype by drought interaction was found—data not shown). However, some differences were observed between genotypes in the size of the effects. For example, dry weight at 34 DAS was lower in the competition treatment under both water treatments, with a uniform response for all lines under the WW condition and more variable under WL (Fig. 2). PI533810, PI533769, and PI534070 had a large difference in response to competition in the WW treatment and almost no difference under WL (Fig. 2a and b). Plant height was lower in the competition treatment for all lines under both water treatments. PI576364 and PI533769 had smaller responses to the presence of neighbours but did show clear responses to the water treatment (Fig. 2c and d). Leaf area was lower in the competition treatment under WL and WW (Fig. 2e and f). The response to competition under WL was variable between lines, with lines PI656025, PI576364, and PI656027 having a large response and PI534070 having the smallest response. Under WW, the response to neighbouring plants was mostly uniform across all lines.

### Experiment 2

#### Irrigation with leachates leads to a response to neighbours without plants growing in the same pot

**Figure 3.**
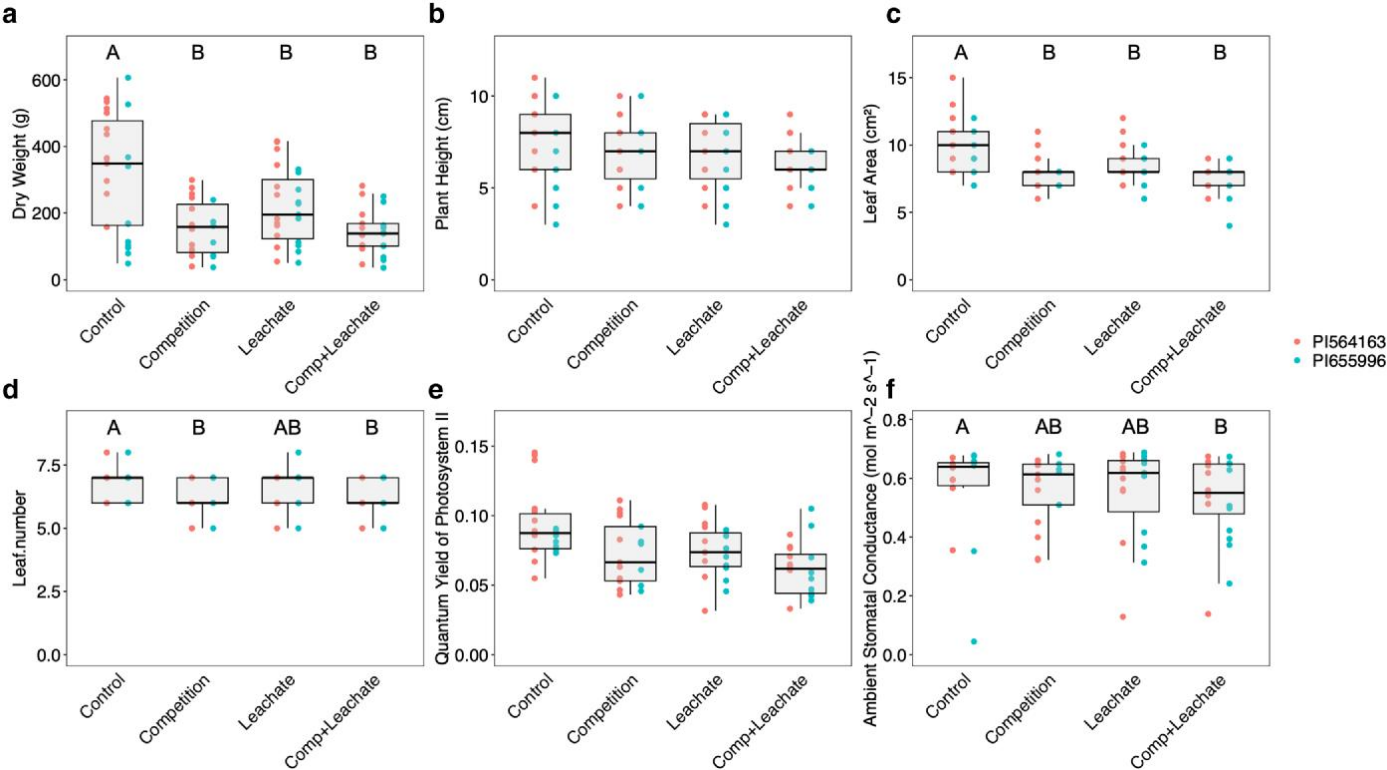
Shoot dry weight (a), plant height (b), leaf area (c), leaf number (d), the quantum yield of PS II (e), and ambient stomatal conductance (f) of sorghum plants of two genotypes grown as a single plant (control) or a plant surrounded by neighbours (competition) and irrigated with a nutrient solution passed through a pot full of media alone (control and competition) or pots with sorghum plants (leachate—including root exudates and leachate + competition). Different letters represent a significant difference between treatments in a Tukey HSD test (*P* < .05).

Based on the reduction in plant productivity in response to the presence of neighbouring plants, even in treatments with ample resources and in the absence of shading, we hypothesized that the signal for the presence of neighbours could be mimicked by transferring root rhizodeposits to pots with individual plants growing in them. Shoot dry weight was significantly (see Supplementary Table S2 for *F* ratios and in-depth details) reduced by irrigating with leachate (Fig. 3a; Supplementary Table S2), while competition without leachate reduced dry weight to a level between the two leachate treatments. Interestingly, plant height was not significantly reduced, albeit plants in all treatments were shorter than the control (Fig. 3b). Leaf area was significantly reduced by irrigating with leachate (Fig. 3c). Leaf number was significantly reduced in both treatments that included competition (Fig. 3d), while the leachate treatment did not show a significant effect. Competition without leachate resulted in a reduction between the two leachate treatments (Fig. 3e). Ambient stomatal conductance (*P* = .0023) and PSII activity (not significant) were reduced only when comparing the leachate + competition treatment to the control, with both the leachate and competition treatments in between the two (Fig. 3e and f). Notably, the competition and leachate were mostly similar, and the combination of both did not result in a cumulative decrease, perhaps pointing to a saturation of neighbour response. Between the two lines selected for this experiment, PI564163 (BTx623) produced plants with significantly higher shoot and root dry weight, leaf area, and height (Supplementary Figure S2). However, no significant interaction between genotype and competition/leachate was found.

**Figure 4.**
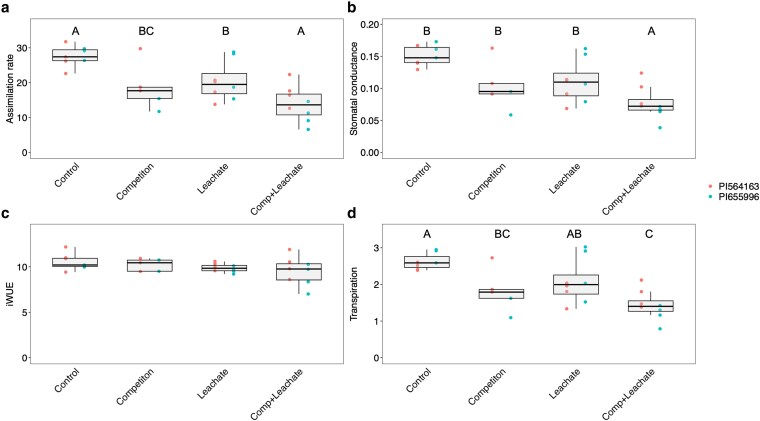
Steady-state assimilation rate (µmol CO_2_ m^−2^ s^−1^) (a), stomatal conductance (mol m^−2^ s^−1^) (b), instantaneous water-use efficiency (iWUE, µmol CO_2_ m^−2^ s^−1^/mmol H_2_O m^−2^ s^−1^) (c), and transpiration rate (mmol m^−2^ s^−1^) (d) of sorghum plants of two genotypes grown as a single plant or a plant surrounded by neighbours and irrigated with a nutrient solution passed through a pot full of media (control) or pots with sorghum plants growing in the same media (leachate—including root exudates). Different letters represent a significant difference between treatments in Tukey HSD tests (*P* < .05).

**Figure 5.**
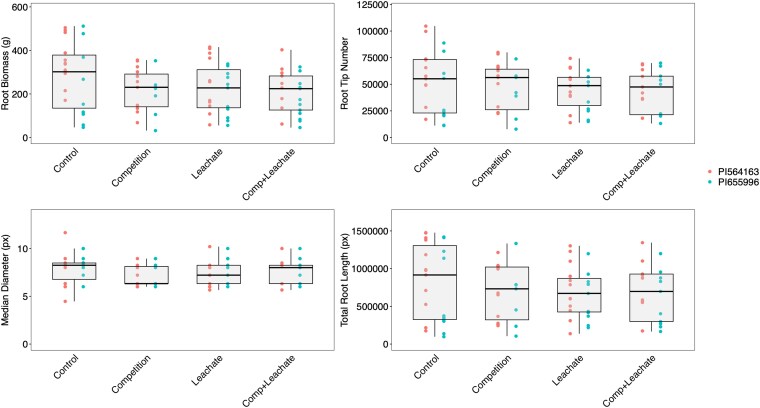
Root biomass (a), number of root tips (b), median root diameter (c), and total root length (d) of sorghum plants of two genotypes grown as a single plant or a plant surrounded by neighbours and irrigated with a nutrient solution passed through a pot full of media (control) or pots with sorghum plants growing in the same media (leachate—including root exudates). Root architecture analysis was conducted using the RhizoVision Explorer v2.0.3 software on images scanned at 600 dpi.

Steady-state photosynthesis measurements were taken to test the changes in photosynthetic ability under different treatments (Fig. 4). Our measurements show that assimilation rate, stomatal conductance, and transpiration rate were all significantly reduced under the leachate treatment compared to the control and further significantly lowered under leachate + competition, with the competition (no leachate) treatment in between the two (Fig. 4a, b, and d). Instantaneous water-use efficiency was unaffected by any treatments (Fig. c). We found a trend of competition and/or leachate reducing root diameter, root tip number, and total root length, but this effect was not statistically significant (Fig. 5). The results in all measured root traits were consistent, albeit not statistically significant. Root traits showed high variability among individuals, suggesting a greater sample size would be required to conclusively demonstrate the effects of neighbours and leachate on root architecture.

## Discussion

Through two complementary experiments, we sought to investigate whether the belowground effects of neighbours could change plant growth, development, and physiology, even in the presence of ample resources. Overall, we found support for our hypothesis that responses to neighbours’ roots can influence plant productivity independent of nutrient availability. Our findings also support a stronger effect under drought. In EXP 1, neighbouring roots’ physical presence reduced sorghum height and biomass to levels similar to plants grown under drought despite sufficient water and nutrients (Fig. 1, Supplementary Table S1). These results contradict our prediction that plants would grow taller in response to neighbours to enhance light capture, as would be expected when shade is present (Earley et al. 1966). Instead, the observed reductions in productivity, even under non-limiting conditions, align with previous findings in both maize (Sarlangue et al. 2007) and sorghum (Jahanzad et al. 2013), where increased planting densities negatively impacted yield, suggesting that densities could not be explained by resource competition alone.

To further study the nature of responses found in EXP 1, we used a leachate transfer experiment (EXP 2) to evaluate whether the adverse effects of competing neighbours could be mimicked by compounds in the leachates. The leachate transfer experiment was designed to focus on the belowground communication between plants, which is an important communication channel (Bais et al. 2004, Khashi et al. 2019). This method also allowed us to study the root system of plants grown with and without the presence of signals from neighbouring plants’ roots. The productivity and photosynthetic activity of plants exposed to competitors or leachates were reduced (Figs 3 and 4). The similarity in response between treatments with physical neighbouring plants in EXP 1 and EXP 2 suggests the involvement of root signalling, which would support our hypothesis about the sensing of neighbours occurring belowground. However, we cannot fully exclude other shared stress or physiological pathways contributing to the observed effects. The response to irrigation with leachate seems to indicate the existence of a root or rhizosphere-based biochemical response independent across varying growing systems and water availabilities and under very high levels of fertilizers (Figs 2 and 3). Our findings suggest that under drought (present in EXP 1), the effect of neighbours leads to larger stress compared to drought without competition treatments (Fig. 1), supporting our hypotheses about a larger response to neighbours under limited resources.

Several mechanisms may explain the decrease in plant productivity we observed in response to neighbours. First, root-root signalling could occur via the transfer of plant hormones. One example of a response to plant hormones is cytokinin sensing (Bollmark and Eliasson 1986), which is present in root exudates and taken up by roots (Soejima et al. 1992, Zahir et al. 2001). Further, the movement of cytokinin from root to shoot can cause changes in stomatal conductance and productivity (Glanz-Idan et al. 2020). We also found a non-significant trend of competitor leachates affecting root architectural traits, leading to smaller root systems with fewer tips (Fig. 5). Given the non-significant nature of this trend, we interpret this result with caution and encourage future work to confirm whether this effect is reproducible under different conditions. Another plant hormone potentially important to belowground signalling is SLs (Al-Babili and Bouwmeester 2015). Studies in other species, including grasses, have shown that belowground biochemical signalling can reduce plant productivity. For example, a study of rice (*Oryza sativa*) showed that plants produced fewer tillers in response to neighbours producing active SLs in the growth media and that mutants in the D14 receptor of SL did not respond to neighbours. SLs have also been reported to decrease stomatal conductance and lead to higher *δ*^13^C in *Arabidopsis* plants (Scaffidi et al. 2014, Lorts and Lasky 2020).

A second potential mechanism for plant–plant interaction in our system is the autotoxicity of allelopathic compounds. Studies have found that a major allelopathic molecule in sorghum exudates, sorgoleone, can lead to reductions in PSII activity and carbon assimilation rate (Einhellig et al. 1993, Nimbal et al. 1996, Cheema et al. 2002, Uddin et al. 2012). These studies used concentrated forms of sorgoleone extracted in organic solvents and applied at very high concentrations (10–200 µM), sometimes directly on leaves. Similarly, studies that tested the effect of sorgoleone on root water uptake found a reduction at high concentrations in seedlings (Hejl and Koster 2004). Negative impacts of sorgoleone have been reported in certain sorghum varieties, with concentrations of 10%–33% of sorgoleone solution in distilled water applied to seedlings eliciting an almost linear response (Tibugari et al. 2020). However, subsequent research indicated that sorgoleone does not translocate effectively within the plant and primarily impacts seedlings (Dayan et al. 2009). Notably, autotoxicity has only been demonstrated under these unusually high concentrations. Our study found no evidence of an enhanced response in the competition + leachate treatment compared to the individual competition or leachate treatments. Given the reported linear response to sorgoleone, even under very high concentrations, such a response would have been expected. Hence, we do not think sorgoleone was solely responsible for our findings. The lower importance of sorgoleone autotoxicity in sorghum plants is strengthened by the targeting of higher concentrations of sorgoleone in breeding programmes (Maharjan et al. 2024), presumably without major autotoxicity. Still, we cannot entirely rule out that sorgoleone, or an interaction with other compounds, may have played a role in response to neighbours and leachates. Low-level autotoxic effects of sorgoleone or synergistic interactions with other compounds may have played a role. Further, sorgoleone and other compounds in sorghum exudate (Cheema et al. 2013) could be the metabolites sensed by the target plants in our study (apart from any toxicity).

By adding ample soil nutrients and, in some treatments, sufficient water, we attempted to remove any soil resource limitation as a potential mechanism affecting the plant–plant interactions within our study. However, we cannot rule out that nutrient depletion may have negatively impacted the growth of our target plants in either EXP 1 or 2. We used very high fertilization rates in both experiments, including irrigating all source pots with nutrient solutions every 2 days in the leachate transfer experiment. Yet a recent study (Paporisch et al. 2021) showed improved retention of nutrients in the soil in the presence of exudates. It could, therefore, be possible that the high nutrient application rate was accompanied by higher retention in the source pots of EXP 2, thereby reducing nutrient availability to our target plants.

Finally, the transfer of microbes and their metabolites represents a possible pathway for indirect plant–plant interactions, which leachates could carry. In this study, pots were filled with the same new media, and the plant–plant interaction occurred within the same species. We do not know of studies that presented evidence of pathological microbes transferring between healthy plants, and we do not expect differences in the microbial community between the source and target pots. Thus, we do not believe the decreased performance is primarily an effect of microbial transfer between pots. However, we cannot rule out some effects of the soil microbiome, including changes in microbial activity.

While we cannot rule out or assign specific values to the effects that the above-mentioned types of interaction may have had on our results, we believe our results support our hypothesis that biochemical compounds (including autotoxic, sorghum allelopathic compounds) transferred in leachates lead to changes in plant development.

The aboveground responses to neighbours (reduced stomatal conductance, reduced size, leaf number—except for the leachate treatment and leaf area) suggest an avoidance strategy when a plant senses neighbours, perhaps limiting their photosynthetic activity and reducing resource demand, which could, in turn, require smaller root systems. A smaller, steeper root system has been shown to contribute to facilitation in multispecies communities (Postma and Lynch 2012). While this could have a negative effect at an individual plant level, this type of strategy may support increased field-level or population productivity and represent a different aspect of increased fitness that might have been selected for in a post-domestication field environment (Lynch 2013). A possible benefit for the individual plant could be limiting its development in response to neighbours and thus reducing its requirements for nutrients and avoiding resource depletion. Selecting for smaller plants can be observed when considering studies pointing to higher productivity under increased densities in sorghum (Habyarimana et al. 2004) and maize (Cox 1996).

## Conclusions

Increasing the productivity of field crops, often achieved through higher planting densities, has been a common strategy since the middle of the last century (Donald 1968), is still a target for modern breeders. Most studies of ideotypes for high-density growing have primarily focused on aboveground phenotypes, including plant–plant interactions like shading. A notable exception is the study by Lynch (2013), which focused on root architecture and anatomy and their importance in an ideotype for high-density growing. Lynch (2013) recommended selecting steep root architectures, which reduce competition. Further, most ideotypes target stand-level productivity and do not focus on individual plants and their responses to the environment.

The results reported here, obtained in systems that allowed for plant–plant interactions to occur belowground, suggest that plant breeding should also include evaluating a plant’s effect on, and potential to be affected by, the presence of conspecific neighbours, including via belowground signals. The variation in response to belowground sensing neighbours found here represents a possible tool for studying these responses. Breeders can possibly identify plants less responsive to rhizodeposits and belowground signals. Less responsive plants, which uptake nutrients and resources at a similar rate regardless of density, could be better suited for extremely high densities in high-input environments. While our findings suggest potential applications in breeding programmes, the underlying mechanisms of belowground signalling remain only partially understood. Additional research is necessary to determine whether such responses are consistent across genotypes, species, and field environments. Transferring rhizodeposits allows researchers and breeders to isolate and simulate belowground interaction under standardized conditions. This method can inform the selection of genotypes that perform well under competitive environments without confounding effects from aboveground interactions.

## Supplementary Material

plaf065_Supplementary_Data

## Data Availability

All raw data are available at Ben Zeev, S. (2025). Neighbor sensing through rhizodeposits in sorghum affects plant physiology and productivity [Data set]. Zenodo. https://doi.org/10.5281/zenodo.16973674.

## References

[plaf065-B1] Afifi M, Swanton C. Maize seed and stem roots differ in response to neighbouring weeds: maize roots differ in response to weeds. Weed Res 2011;51:442–50. 10.1111/j.1365-3180.2011.00865.x

[plaf065-B2] Ahlmann-Eltze C, Patil I. ggsignif: R package for displaying significance brackets for ‘ggplot2’. PsyArXiv, 10.31234/osf.io/7awm6, 8 April 2021, preprint: not peer reviewed.

[plaf065-B3] Al-Babili S, Bouwmeester HJ. Strigolactones, a novel carotenoid-derived plant hormone. Annu Rev Plant Biol 2015;66:161–86. 10.1146/annurev-arplant-043014-114759 25621512

[plaf065-B4] Aschehoug ET, Brooker R, Atwater DZ et al The mechanisms and consequences of interspecific competition among plants. Annu Rev Ecol Evol Syst 2016;47:263–81. 10.1146/annurev-ecolsys-121415-032123

[plaf065-B5] Babiker A, Hamdoun A. Factors affecting the activity of ethephon in stimulating seed germination of *Striga hermonthica* (Del.) Benth. Weed Res 1983;23:125–31. 10.1111/j.1365-3180.1983.tb00530.x

[plaf065-B6] Badri DV, De-la-Peña C, Lei Z et al Root secreted metabolites and proteins are involved in the early events of plant–plant recognition prior to competition. PLoS One 2012;7:e46640. 10.1371/journal.pone.0046640 23056382 PMC3462798

[plaf065-B7] Bais HP, Park S-W, Weir TL et al How plants communicate using the underground information superhighway. Trends Plant Sci 2004;9:26–32. 10.1016/j.tplants.2003.11.008 14729216

[plaf065-B8] Bellis ES, Kelly EA, Lorts CM et al Genomics of sorghum local adaptation to a parasitic plant. Proc Natl Acad Sci 2020;117:4243–51. 10.1073/pnas.1908707117 32047036 PMC7049153

[plaf065-B9] Berenguer M, Faci J. Sorghum (*Sorghum bicolor* L. Moench) yield compensation processes under different plant densities and variable water supply. Eur J Agron 2001;15:43–55. 10.1016/S1161-0301(01)00095-8

[plaf065-B10] Bhowmik P, Doll J. Allelopathic effects of annual weed residues on growth and nutrient uptake of corn and soybeans. Agron J 1984;76:383–8. 10.2134/agronj1984.00021962007600030008x

[plaf065-B11] Bilas RD, Bretman A, Bennett T. Friends, neighbours and enemies: an overview of the communal and social biology of plants. Plant Cell Environ 2021;44:997–1013. 10.1111/pce.13965 33270936

[plaf065-B12] Bogatek R, Gniazdowska A, Zakrzewska W et al Allelopathic effects of sunflower extracts on mustard seed germination and seedling growth. Biol Plant 2006;50:156–8. 10.1007/s10535-005-0094-6

[plaf065-B13] Bollmark M, Eliasson L. Effects of exogenous cytokinins on root formation in pea cuttings. Physiol Plant 1986;68:662–6. 10.1111/j.1399-3054.1986.tb03414.x

[plaf065-B14] Callaway RM, Walker LR. Competition and facilitation: a synthetic approach to interactions in plant communities. Ecology 1997;78:1958–65. 10.1890/0012-9658(1997)078[1958:CAFASA]2.0.CO;2

[plaf065-B15] Casper BB, Jackson RB. Plant competition underground. Annu Rev Ecol Syst 1997;28:545–70. 10.1146/annurev.ecolsys.28.1.545

[plaf065-B16] Cheema ZA, Farooq M, Wahid A (Eds.). Allelopathy: Current Trends and Future Applications. Berlin: Springer; 2013.

[plaf065-B17] Cheema ZA, Iqbal M, Ahmad R. Response of wheat varieties and some rabi weeds to allelopathic effects of sorghum water extract. Int J Agric Biol 2002;4:52–5.

[plaf065-B18] Cheema ZA, Khaliq A. Use of sorghum allelopathic properties to control weeds in irrigated wheat in a semi arid region of Punjab. Agric Ecosyst Environ 2000;79:105–12. 10.1016/S0167-8809(99)00140-1

[plaf065-B19] Cox WJ . Whole-plant physiological and yield responses of maize to plant density. Agron J 1996;88:489–96. 10.2134/agronj1996.00021962008800030022x

[plaf065-B20] Dayan FE, Howell J, Weidenhamer JD. Dynamic root exudation of sorgoleone and its in planta mechanism of action. J Exp Bot 2009;60:2107–17. 10.1093/jxb/erp082 19357432 PMC2682501

[plaf065-B21] De Wet J, Harlan J. The origin and domestication of *Sorghum bicolor*. Econ Bot 1971;25:128–35. 10.1007/BF02860074

[plaf065-B22] Donald CM . The breeding of crop ideotypes. Euphytica 1968;17:385–403. 10.1007/BF00056241

[plaf065-B23] Du X, Wang Z, Lei W et al Increased planting density combined with reduced nitrogen rate to achieve high yield in maize. Sci Rep 2021;11:358. 10.1038/s41598-020-79633-z 33432054 PMC7801644

[plaf065-B24] Duchene O, Vian J-F, Celette F. Intercropping with legume for agroecological cropping systems: complementarity and facilitation processes and the importance of soil microorganisms. A review. Agric Ecosyst Environ 2017;240:148–61. 10.1016/j.agee.2017.02.019

[plaf065-B25] Earley EB, Miller RJ, Reichert GL et al Effect of shade on maize production under field conditions. Crop Sci 1966;6:1–7. 10.2135/cropsci1966.0011183X000600010001x

[plaf065-B26] Einhellig FA, Rasmussen JA, Hejl AM et al Effects of root exudate sorgoleone on photosynthesis. J Chem Ecol 1993;19:369–75. 10.1007/BF00993702 24248881

[plaf065-B27] Einhellig FA, Souza IF. Phytotoxicity of sorgoleone found in grain Sorghum root exudates. J Chem Ecol 1992;18:1–11. 10.1007/BF00997160 24254628

[plaf065-B28] Falik O, Hoffmann I, Novoplansky A. Say it with flowers: flowering acceleration by root communication. Plant Signal Behav 2014;9:e28258. 10.4161/psb.28258 24598343 PMC4091325

[plaf065-B29] Farquhar G, Richards R. Isotopic composition of plant carbon correlates with water-use efficiency of wheat genotypes. Funct Plant Biol 1984;11:539–52. 10.1071/PP9840539

[plaf065-B30] Farrior CE . Competitive optimization models, attempting to understand the diversity of life. New Phytol 2014;203:1025–7. 10.1111/nph.12940 25077786

[plaf065-B31] Gerik TJ, Neely CL. Plant density effects on main culm and tiller development of grain Sorghum. Crop Sci 1987;27:1225–30. 10.2135/cropsci1987.0011183X002700060027x

[plaf065-B32] Glanz-Idan N, Tarkowski P, Turečková V et al Root–shoot communication in tomato plants: cytokinin as a signal molecule modulating leaf photosynthetic activity. J Exp Bot 2020;71:247–57. 10.1093/jxb/erz399 31504736 PMC6913696

[plaf065-B33] Habyarimana E, Bonardi P, Laureti D et al Multilocational evaluation of biomass sorghum hybrids under two stand densities and variable water supply in Italy. Ind Crops Prod 2004;20:3–9. 10.1016/j.indcrop.2003.12.020

[plaf065-B34] Hejl AM, Koster KL. The allelochemical sorgoleone inhibits root H^+^-ATPase and water uptake. J Chem Ecol 2004;30:2181–91. 10.1023/B:JOEC.0000048782.87862.7f 15672664

[plaf065-B35] Henriksson N, Franklin O, Tarvainen L et al The mycorrhizal tragedy of the commons. Ecol Lett 2021;24:1215–24. 10.1111/ele.13737 33749095

[plaf065-B36] Hilhorst HWM, Karssen CM. Effect of chemical environment on seed germination. In: Fenner M (ed.) Seeds: The Ecology of Regeneration in Plant Communities. UK: CABI Publishing, 2000, 293–309.

[plaf065-B37] Jahanzad E, Jorat M, Moghadam H et al Response of a new and a commonly grown forage sorghum cultivar to limited irrigation and planting density. Agric Water Manag 2013;117:62–9. 10.1016/j.agwat.2012.11.001

[plaf065-B39] Khashi U, Rahman M, Zhou X et al The role of root exudates, CMNs, and VOCs in plant–plant interaction. J Plant Interact 2019;14:630–6. 10.1080/17429145.2019.1689581

[plaf065-B40] Kuznetsova A, Brockhoff PB, Christensen RHB. Package ‘lmertest’. R package version 2015;2:734.

[plaf065-B41] Lenth R, Lenth MR. Package ‘lsmeans.’. Am Stat 2018;34:216–21.

[plaf065-B42] Lepik A, Abakumova M, Davison J et al Spatial mapping of root systems reveals diverse strategies of soil exploration and resource contest in grassland plants. J Ecol 2021;109:652–63. 10.1111/1365-2745.13535

[plaf065-B43] Lorts CM, Lasky JR. Competition × drought interactions change phenotypic plasticity and the direction of selection on Arabidopsis traits. New Phytol 2020;227:1060–72. 10.1111/nph.16593 32267968

[plaf065-B44] Lynch JP . Steep, cheap and deep: an ideotype to optimize water and N acquisition by maize root systems. Ann Bot 2013;112:347–57. 10.1093/aob/mcs293 23328767 PMC3698384

[plaf065-B45] Maharjan B, Leon F, Rooney WL et al Combining abilities and quantitative inheritance of sorgoleone exudation in *Sorghum bicolor*. Crop Sci 2024;64:3219–3230. 10.1002/csc2.21366

[plaf065-B46] Mwangangi IM, Büchi L, Runo S et al Essential plant nutrients impair post-germination development of Striga in sorghum. Plants People Planet 2023;7:422–435. 10.1002/ppp3.10418

[plaf065-B47] Nimbal CI, Pedersen JF, Yerkes CN et al Phytotoxicity and distribution of sorgoleone in grain sorghum germplasm. J Agric Food Chem 1996;44:1343–7. 10.1021/jf950561n

[plaf065-B48] Page ER, Liu W, Cerrudo D et al Shade avoidance influences stress tolerance in maize. Weed Sci 2011;59:326–34. 10.1614/WS-D-10-00159.1

[plaf065-B49] Paporisch A, Bavli H, Strickman RJ et al Root exudates alters nutrient transport in soil. Water Resour Res 2021;57:e2021WR029976. 10.1029/2021WR029976

[plaf065-B50] Pardo J, VanBuren R. Evolutionary innovations driving abiotic stress tolerance in C4 grasses and cereals. Plant Cell 2021;33:3391–401. 10.1093/plcell/koab205 34387354 PMC8566246

[plaf065-B51] Pennsylvania State University, Agricultural Extension Service . Penn State extension service fertilizer recommendation table. Pennsylvania State University.

[plaf065-B52] Postma JA, Lynch JP. Complementarity in root architecture for nutrient uptake in ancient maize/bean and maize/bean/squash polycultures. Ann Bot 2012;110:521–34. 10.1093/aob/mcs082 22523423 PMC3394648

[plaf065-B53] Proust H, Hoffmann B, Xie X et al Strigolactones regulate protonema branching and act as a quorum sensing-like signal in the moss *Physcomitrella patens*. Development 2011;138:1531–9. 10.1242/dev.058495 21367820

[plaf065-B54] Putnam AR . Allelopathic Research in Agriculture: Past Highlights and Potential. ACS Publications, 1985.

[plaf065-B55] Raphaël P, Kakkera A, Jana K et al The genotypic variation in the positive response of sorghum to higher sowing density is linked to an increase in water use efficiency. Eur J Agron 2024;158:127207. 10.1016/j.eja.2024.127207

[plaf065-B56] Raza T, Yahya Khan M, Mahmood Nadeem S et al Biological management of selected weeds of wheat through co-application of allelopathic rhizobacteria and sorghum extract. Biol Control 2021;164:104775. 10.1016/j.biocontrol.2021.104775

[plaf065-B71] R Core Team. *R: A Language and Environment for Statistical Computing*. Vienna, Austria: R Foundation for Statistical Computing, 2025. https://www.R-project.org/.

[plaf065-B57] Sandhu S, Dhillon BS. Breeding plant type for adaptation to high plant density in tropical maize—a step towards productivity enhancement. Plant Breed 2021;140:509–18. 10.1111/pbr.12949

[plaf065-B58] Sarlangue T, Andrade FH, Calviño PA et al Why do maize hybrids respond differently to variations in plant density? Agron J 2007;99:984–91. 10.2134/agronj2006.0205

[plaf065-B59] Scaffidi A, Waters MT, Sun YK et al Strigolactone hormones and their stereoisomers signal through two related receptor proteins to induce different physiological responses in Arabidopsis. Plant Physiol 2014;165:1221–32. 10.1104/pp.114.240036 24808100 PMC4081333

[plaf065-B60] Schenk HJ . Root competition: beyond resource depletion. J Ecol 2006;94:725–39. 10.1111/j.1365-2745.2006.01124.x

[plaf065-B61] Schmid C, Bauer S, Müller B et al Belowground neighbor perception in Arabidopsis thaliana studied by transcriptome analysis: roots of *Hieracium pilosella* cause biotic stress. Front Plant Sci 2013;4:296. 10.3389/fpls.2013.00296 23967000 PMC3743015

[plaf065-B62] Seethepalli A, Dhakal K, Griffiths M et al RhizoVision Explorer: open-source software for root image analysis and measurement standardization. AoB Plants 2021;13:plab056. 10.1093/aobpla/plab056 34804466 PMC8598384

[plaf065-B63] Seethepalli A, York LM. RhizoVision Explorer-Interactive software for generalized root image analysis designed for everyone (Version 2.0. 2). Zenodo. 2020.

[plaf065-B64] Soejima H, Sugiyama T, Ishihara K. Changes in cytokinin activities and mass spectrometric analysis of cytokinins in root exudates of rice plant (*Oryza sativa* L.): comparison between cultivars Nipponbare and Akenohoshi. Plant Physiol 1992;100:1724–9. 10.1104/pp.100.4.1724 16653189 PMC1075856

[plaf065-B65] Tibugari H, Chiduza C, Mashingaidze A et al High sorgoleone autotoxicity in sorghum (*Sorghum bicolor* (L.) Moench) varieties that produce high sorgoleone content. S Afr J Plant Soil 2020;37:160–7. 10.1080/02571862.2020.1711539

[plaf065-B66] Uddin M, Park K, Han S et al Effects of sorgoleone allelochemical on chlorophyll fluorescence and growth inhibition in weeds. Allelopathy J 2012;30:61–70.

[plaf065-B67] Wei T, Simko V, Levy M et al Package ‘corrplot’. Statistician 2017;56:e24.

[plaf065-B68] Wheeldon CD, Hamon-Josse M, Lund H et al Environmental strigolactone drives early growth responses to neighboring plants and soil volume in pea. Curr Biol 2022;32:3593–3600.e3. 10.1016/j.cub.2022.06.063 35839764 PMC9616727

[plaf065-B69] Wickham H, Chang W, Wickham MH. Package ‘ggplot2.’ Create elegant data visualisations using the grammar of graphics. Version 2: 1-189. 2016.

[plaf065-B70] Zahir ZA, Asghar HN, Arshad M. Cytokinin and its precursors for improving growth and yield of rice. Soil Biol Biochem 2001;33:405–8. 10.1016/S0038-0717(00)00145-0

