# Supplementary material for: Neighbour sensing through rhizodeposits in sorghum affects plant physiology and productivity
Source: AoB Plants. 2025 Nov 13;17(6):plaf065. doi: 10.1093/aobpla/plaf065 (PMC12672025; doi:10.1093/aobpla/plaf065)
Supplement: plaf065_Supplementary_Data [file plaf065_supplementary_data.zip › Table S1.docx]

Table S1: Sorghum landraces used in the experiment.

| Line code | PI | Name | Origin | Remarks from USDA grin/ sorghum collection (where applicable) |
| --- | --- | --- | --- | --- |
| L1 | PI 655996 | RTx430 | Texas | vegetative drought tolerance, natural LGS1 LOF |
| L2 | PI565121 | Macia | Zimbabwe | background of LGS1 mutants |
| L3 | PI564163 | BTx623 | Texas | Pre-flowering drought tolerant |
| L4 | PI656025 | Shanqui Red | China | high striga germination |
| L5 | PI533810 | Karad 2-7-11 | India | Plants short-statured, Photoperiod insensitive |
| L6 | PI576364 | Chari Uri | India | Fertility reaction Maintainer |
| L7 | PI656027 | SRN-39 | Sudan | low striga germination |
| L8 | PI533752 | SC103 | South Africa | low striga germination, short-statured, Photoperiod insensitive |
| L9 | PI533769 | 290 Feterita Shendi 2 | Sudan | Plants short-statured, Photoperiod insensitive. |
| L10 | PI534070 | BE 25 | Nigeria | Plants short-statured, Photoperiod insensitive. |
