# Supplementary material for: Neighbour sensing through rhizodeposits in sorghum affects plant physiology and productivity
Source: AoB Plants. 2025 Nov 13;17(6):plaf065. doi: 10.1093/aobpla/plaf065 (PMC12672025; doi:10.1093/aobpla/plaf065)
Supplement: plaf065_Supplementary_Data [file plaf065_supplementary_data.zip › TableS2_BenZeev_Formatted_10.4.docx]

Table S2: Sources of variance and significance in Experiment 1 (top section) and Experiment 2 (bottom three sections ANOVA results. Genotype and treatment main effects are presented along with the Tukey HSD significance test (F ratio) and P values.

| Trait | Source | NumDF | DenDF | F value | P value | Signif. |
| --- | --- | --- | --- | --- | --- | --- |
| EXP 1: | | | | | | |
| Dry_Weight..g. | Genotype | 9 | 110 | 2.9904 | 0.003175 | ** |
| Dry_Weight..g. | Comp | 1 | 110 | 44.5732 | 1.04E-09 | *** |
| Dry_Weight..g. | Drought | 1 | 110 | 107.4055 | 2.20E-16 | *** |
| Dry_Weight..g. | Genotype:Comp | 9 | 110 | 0.7045 | 0.703565 |  |
| Dry_Weight..g. | Genotype:Drought | 9 | 110 | 1.3779 | 0.206749 |  |
| Dry_Weight..g. | Comp:Drought | 1 | 110 | 9.6235 | 0.002441 | ** |
| Dry_Weight..g. | Genotype:Comp:Drought | 9 | 110 | 0.4563 | 0.900628 |  |
| Leaf.area..cm.sq. | Genotype | 9 | 111 | 1.3549 | 0.2174 |  |
| Leaf.area..cm.sq. | Comp | 1 | 111 | 60.1628 | 4.57E-12 | *** |
| Leaf.area..cm.sq. | Drought | 1 | 111 | 106.9756 | 2.20E-16 | *** |
| Leaf.area..cm.sq. | Genotype:Comp | 9 | 111 | 0.1755 | 0.9962 |  |
| Leaf.area..cm.sq. | Genotype:Drought | 9 | 111 | 1.0438 | 0.4103 |  |
| Leaf.area..cm.sq. | Comp:Drought | 1 | 111 | 21.6281 | 9.19E-06 | *** |
| Leaf.area..cm.sq. | Genotype:Comp:Drought | 9 | 111 | 0.4901 | 0.8786 |  |
| Stomatal_Conductance..mol.1m.2s.1. | Genotype | 9 | 111 | 1.0305 | 0.4205 |  |
| Stomatal_Conductance..mol.1m.2s.1. | Comp | 1 | 111 | 5.5533 | 0.0202 | * |
| Stomatal_Conductance..mol.1m.2s.1. | Drought | 1 | 111 | 1.4574 | 0.2299 |  |
| Stomatal_Conductance..mol.1m.2s.1. | Genotype:Comp | 9 | 111 | 1.3589 | 0.2155 |  |
| Stomatal_Conductance..mol.1m.2s.1. | Genotype:Drought | 9 | 111 | 0.8545 | 0.568 |  |
| Stomatal_Conductance..mol.1m.2s.1. | Comp:Drought | 1 | 111 | 0.1908 | 0.6631 |  |
| Stomatal_Conductance..mol.1m.2s.1. | Genotype:Comp:Drought | 9 | 111 | 0.781 | 0.6342 |  |
| Quantum_yield_of_Photosystem_II | Genotype | 9 | 111.3 | 0.9261 | 0.505484 |  |
| Quantum_yield_of_Photosystem_II | Comp | 1 | 111.1 | 9.3635 | 0.002775 | ** |
| Quantum_yield_of_Photosystem_II | Drought | 1 | 111.5 | 0.2681 | 0.605602 |  |
| Quantum_yield_of_Photosystem_II | Genotype:Comp | 9 | 111.3 | 1.0237 | 0.425701 |  |
| Quantum_yield_of_Photosystem_II | Genotype:Drought | 9 | 111.2 | 1.0918 | 0.374612 |  |
| Quantum_yield_of_Photosystem_II | Comp:Drought | 1 | 111.2 | 0.3834 | 0.537034 |  |
| Quantum_yield_of_Photosystem_II | Genotype:Comp:Drought | 9 | 111.4 | 0.4255 | 0.918995 |  |
| Plant.Height..cm. | Genotype | 9 | 112 | 3.5458 | 0.0006655 | *** |
| Plant.Height..cm. | Comp | 1 | 112 | 28.286 | 5.41E-07 | *** |
| Plant.Height..cm. | Drought | 1 | 112 | 29.1596 | 3.78E-07 | *** |
| Plant.Height..cm. | Genotype:Comp | 9 | 112 | 1.2474 | 0.2738099 |  |
| Plant.Height..cm. | Genotype:Drought | 9 | 112 | 0.6023 | 0.7927574 |  |
| Plant.Height..cm. | Comp:Drought | 1 | 112 | 1.1679 | 0.2821627 |  |
| Plant.Height..cm. | Genotype:Comp:Drought | 9 | 112 | 0.5529 | 0.8326027 |  |
| EXP 2: | | | | | | |
| Shoot | Genotype | 1 | 76.05 | 8.5877 | 0.004467 | ** |
| Shoot | Treat | 3 | 76.13 | 11.5398 | 2.57E-06 | *** |
| Shoot | Genotype:Treat | 3 | 76.13 | 1.6497 | 0.184984 |  |
| Root | Genotype | 1 | 76.06 | 8.3371 | 0.005055 | ** |
| Root | Treat | 3 | 76.15 | 2.0489 | 0.114094 |  |
| Root | Genotype:Treat | 3 | 76.15 | 0.8927 | 0.448929 |  |
| Shoot:Root | Genotype | 1 | 76.02 | 0.1751 | 0.6768 |  |
| Shoot:Root | Treat | 3 | 76.09 | 21.6134 | 3.17E-10 | *** |
| Shoot:Root | Genotype:Treat | 3 | 76.09 | 0.5232 | 0.6677 |  |
| Leaf.Area..cm2. | Genotype | 1 | 78 | 4.539 | 0.03628 | * |
| Leaf.Area..cm2. | Treat | 3 | 78 | 10.4621 | 7.35E-06 | *** |
| Leaf.Area..cm2. | Genotype:Treat | 3 | 78 | 1.7768 | 0.15849 |  |
| gsw.mol.1m.2s.1 | Genotype | 1 | 65.13 | 4.1737 | 0.045107 | * |
| gsw.mol.1m.2s.1 | Treat | 3 | 64.92 | 5.4934 | 0.001999 | ** |
| gsw.mol.1m.2s.1 | Genotype:Treat | 3 | 64.99 | 0.2742 | 0.843799 |  |
| Quantum.yield.of.photosystem.ii | Genotype | 1 | 64.21 | 0.2146 | 0.6447 |  |
| Quantum.yield.of.photosystem.ii | Treat | 3 | 64.14 | 0.1529 | 0.9275 |  |
| Quantum.yield.of.photosystem.ii | Genotype:Treat | 3 | 64.19 | 0.7257 | 0.5404 |  |
| Median.Diameter.px | Genotype | 1 | 76.09 | 3.5291 | 0.06413 | . |
| Median.Diameter.px | Treat | 3 | 76.31 | 1.0297 | 0.38431 |  |
| Median.Diameter.px | Genotype:Treat | 3 | 76.31 | 0.0814 | 0.96994 |  |
| Total.Root.Length.px | Genotype | 1 | 76.04 | 4.2332 | 0.03964 | * |
| Total.Root.Length.px | Treat | 3 | 76.09 | 0.6213 | 0.6011 |  |
| Total.Root.Length.px | Genotype:Treat | 3 | 76.09 | 0.2688 | 0.84793 |  |
| Number.of.Root.Tips | Genotype | 1 | 76.08 | 7.1882 | 0.008995 | ** |
| Number.of.Root.Tips | Treat | 3 | 76.17 | 0.4708 | 0.703535 |  |
| Number.of.Root.Tips | Genotype:Treat | 3 | 76.17 | 0.6711 | 0.57241 |  |
| GasEx.A | Genotype | 1 | 20 | 6.3811 | 0.02002 | * |
| GasEx.A | Treatment | 3 | 20 | 3.9811 | 0.02245 | * |
| GasEx.A | Genotype:Treatment | 3 | 20 | 3.9219 | 0,02364 | * |
| GasEx.gsw | Genotype | 1 | 20 | 5.3151 | 0.03198 | * |
| GasEx.gsw | Treatment | 3 | 20 | 3.3858 | 0.03829 | * |
| GasEx.gsw | Genotype:Treatment | 3 | 20 | 3.7470 | 0.02761 | * |
| WUEinstat | Genotype | 1 | 20 | 0.1464 | 0.7061 |  |
| WUEinstat | Treatment | 3 | 20 | 0.5170 | 0.6754 |  |
| WUEinstat | Genotype:Treatment | 3 | 20 | 0.7083 | 0.6754 |  |
| GasEx.Emm | Genotype | 1 | 20 | 6.4365 | 0.01962 | * |
| GasEx.Emm | Treatment | 3 | 20 | 3.1391 | 0.04813 | * |
| GasEx.Emm | Genotype:Treatment | 3 | 20 | 4.8854 | 0.01044 | * |
