# Supplementary material for: Neighbour sensing through rhizodeposits in sorghum affects plant physiology and productivity
Source: AoB Plants. 2025 Nov 13;17(6):plaf065. doi: 10.1093/aobpla/plaf065 (PMC12672025; doi:10.1093/aobpla/plaf065)
Supplement: plaf065_Supplementary_Data [file plaf065_supplementary_data.zip › Supplementary Figures .pdf]

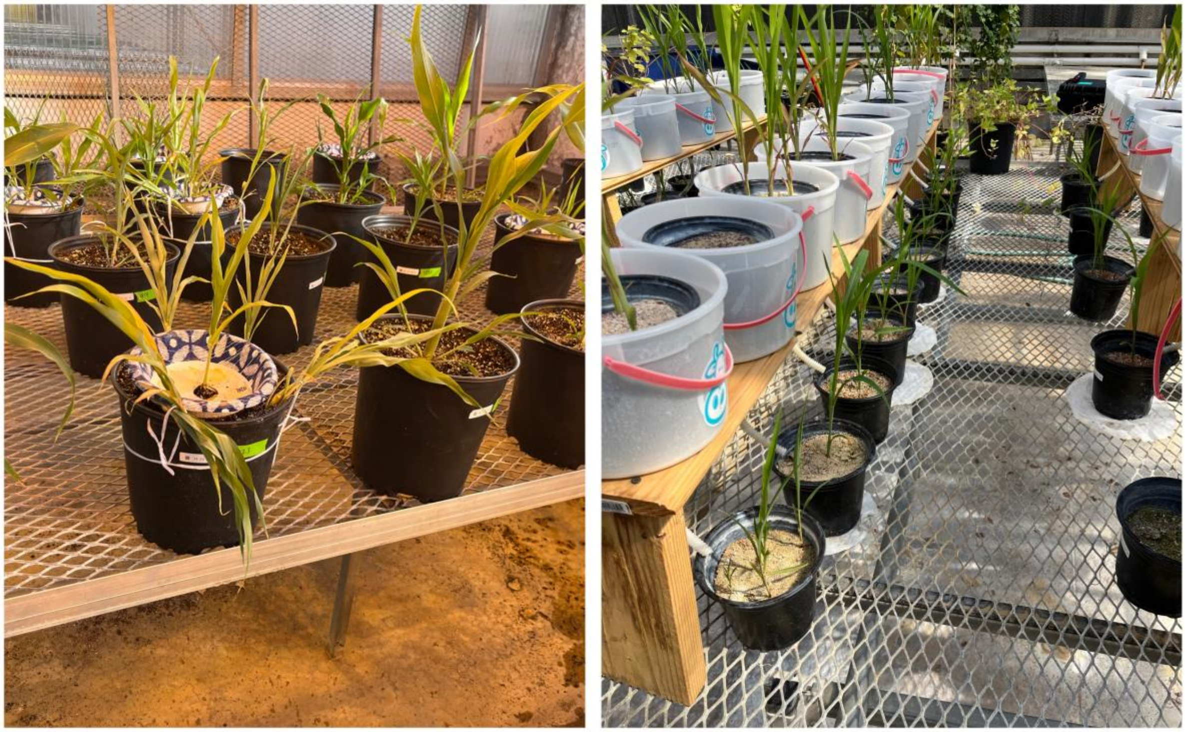

Figure S1: an overview of the experimental setup used for the physical competition experiment (left) and leachate transfer (right).

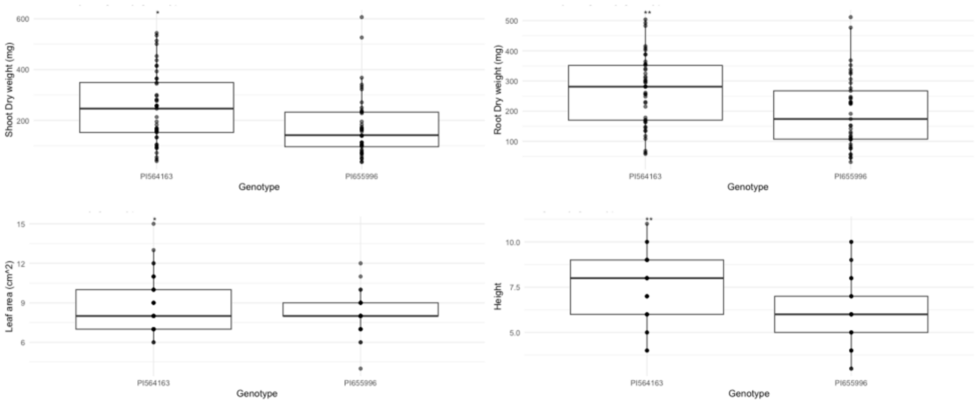

Figure S2: Shoot (top left) and Root (top right) dry weight, Leaf area (bottom left), and plant height (bottom right) of the two sorghum genotypes tested in the leachate transfer experiment (EXP2) - BTx623 and RTx430 (PI564163 and PI655996, respectively). "*", "**", and "***" indicate t- test P values <0.05, <0.01, and <0.001, respectively.
